# “I Am Going to Take It Up”: Implementing Skin‐to‐Skin Contact in Uganda

**DOI:** 10.1111/mcn.70047

**Published:** 2025-05-16

**Authors:** Karin Cadwell, Anna Blair, Kajsa Brimdyr, Kristin Svensson, Melissa Reyes, Mike Kagawa, Louise Racine Bastarache, Scovia Nalugo Mbalinda

**Affiliations:** ^1^ Healthy Children Project, Inc Harwich Massachusetts USA; ^2^ Karolinska Institutet Solna Sweden; ^3^ College of Health Sciences Makerere University Kampala Uganda; ^4^ Harvard Medical Faculty Physicians Cambridge Massachusetts USA

**Keywords:** breastfeeding, implementation, interview, low‐income setting, skin‐to‐skin contact, Uganda

## Abstract

Timely and prolonged skin‐to‐skin contact (SSC) immediately after birth is recommended in the Ugandan Clinical Guideline, the 2023 International Research and Practice Guideline on SSC and by the WHO/UNICEF Baby‐Friendly Hospital Initiative. Skin‐to‐skin contact is safe, low‐resource, evidence‐based and contributes to short‐ and long‐term health outcomes. However, practice is inconsistent. A rapid‐change intervention, PRECESS (Practice, Reflection, Education and training, Combined with Ethnography for Sustainable Success), encouraged adaptation of the SSC Guideline protocol in a regional referral hospital in Uganda. Fifteen key informants, including leadership and staff members, were interviewed before and after the practice change about perceived barriers and solutions for implementing SSC. The semi‐structured pre‐ and post‐intervention interviews were recorded, transcribed and analyzed for meaningful units and themes. Three themes emerged: (1) Commitment to consistent, evidence‐based care within constraints; (2) Addressing knowledge and skill regarding the optimal practice of SSC; and (3) Willingness to “take‐up” the practice change for the benefit of mothers and babies. Our findings support the experiential method of practice change PRECESS to implement immediate, continuous, uninterrupted SSC for at least the first hour after birth. Despite the challenges and barriers identified in key informant interviews, significant progress was made in increasing the duration of SSC for both vaginal and cesarean births. The identified themes provide insight for future implementation of skin‐to‐skin contact.

## Summary

- An experiential intervention significantly increased and sustained the duration of SSC at a regional referral hospital in Uganda.
- Despite constraints of the existing environment, analysis of interviews with staff revealed a commitment to consistent, evidence‐based in‐line with international guidelines.
- Staff expressed a willingness to “take‐up” and sustain the practice change for the benefit of mothers and babies.

## Graphical abstract

Skin‐to‐skin contact (SSC) immediately after birth is recommended clinical practice. This qualitative study used semi‐structured interviews pre‐ and post‐intervention in a hospital in Uganda. Three themes emerged: commitment to consistent, evidence‐based care within constraints; addressing knowledge and skill; and willingness to “take‐up” SSC for the benefit of mothers and babies.

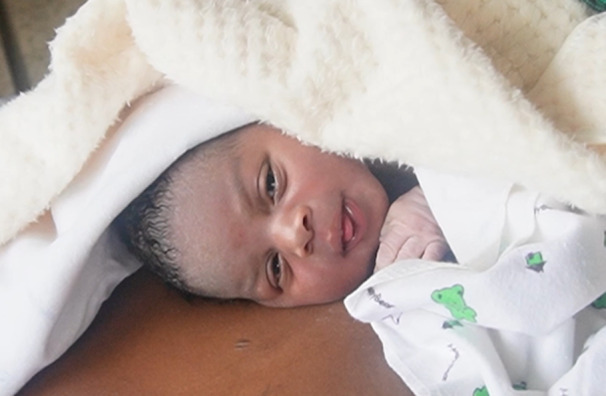

## 1 Introduction

Caring for newborns and their mothers while skin‐to‐skin during the first hour after birth is an evidence‐based practice (Brimdyr et al. 2023) and is strongly recommended by WHO and UNICEF as a key component of the Baby Friendly Hospital Initiative (World Health Organization WHO and the United Nations Children's Fund UNICEF 2020). Immediate, continuous, and uninterrupted skin‐to‐skin contact (SSC) is the standard of care following all modes of birth, for all mothers and babies from 1000 g, (with experienced staff if assistance is needed). When skin‐to‐skin for at least 1 h after birth, the newborn infant progresses through Widstrӧm's 9 Instinctive Stages (A. Widström et al. 2019; A.‐M. Widström et al. 2011) increasing the possibility of self‐attachment and suckling in the first hours after birth (Brimdyr et al. 2018, 2020; Cadwell et al. 2018; Crenshaw et al. 2012; Svensson et al. 2020).

Uninterrupted SSC during this sensitive period after birth (A. Widström et al. 2019) is safe, low resource, cost‐effective, evidence‐based, and contributes to both short‐ and long‐term health outcomes (Moore et al. 2016). Researchers have reported that infant transition indicators such as temperature, blood glucose levels, and oxygen saturation are more optimal when newborns are held in SSC compared to other warming mechanisms such as the use of a radiant warmer (Moore et al. 2016). Full‐term, prematurely born and low birth weight infants transition well in SSC (Linnér et al. 2022). In the case of cesarean birth, immediate SSC supports the safe transition of the newborn (Crenshaw et al. 2019) and SSC decreases the “stress of being born” after vaginal birth (Bystrova et al. 2003). The earliest studies of SSC included only vaginal births and demonstrated a positive relationship to breastfeeding exclusivity and breastfeeding for longer durations (Marín Gabriel et al. 2010; Mikiel‐Kostyra et al. 2002; Mizuno et al. 2004). Recently researchers have reported that mothers who had the experience of SSC had decreased infant‐focused anxiety (Mazúchová et al. 2020) and maternal satisfaction with midwifery and doula care (Liu et al. 2021) and that SSC contact “alleviated the pain of perineal laceration repair and episiotomy suture, shortened the operation time of episiotomy suture and improved the cooperation rate of parturients receiving suture” (Zou et al. 2022). For the mother, comfort levels and oxytocin levels were increased and physiologic measurements of stress were decreased if the newborn was held skin‐to‐skin in the interoperative period after cesarean (Frederick et al. 2020).

SSC has been implemented as an element of different multifocal international strategies, including the Ananya program (Darmstadt et al. 2020), Helping Babies Survive (Dol et al. 2018), the 6‐step intervention package (Mbalinda et al. 2018) and Early Essential Newborn Care (Peven et al. 2020; Qu et al. 2020). Quality improvement/management projects have also been used as a mechanism to improve of the practice SSC (Boyd 2017; Das et al. 2021; Kaushal et al. 2022; LeBlanc et al. 2018), as have education and training programs (Callaghan‐Koru et al. 2016; Caponero et al. 2022; Fritz et al. 2017; Nissen et al. 2019). The experiential intervention, PRECESS (Practice, Reflection, Education and training, Combined with Ethnography for Sustainable Success), used in this study, has been shown to increase the practice of SSC for the first hour after birth in both high‐ and low‐resource countries (Brimdyr et al. 2012; Crenshaw et al. 2012).

The most recent Cochrane Review of early skin‐to‐skin care for full‐term newborns reported that “SSC was defined in various ways and different scales and times were used to measure different outcomes” (Moore et al. 2016) (p. 3). The types of interventions are also a challenge, as Moran acknowledges that most SSC interventions do not succeed at achieving the WHO standard of immediate, continuous, uninterrupted SSC through the first breastfeed (Moran et al. 2022). Acknowledging this dissimilitude, we sought to effectuate the usual practice of SSC in a referral and teaching hospital in Uganda. Initially, clinical practice consisted of SSC immediately after birth and continuing only until the umbilical cord was clamped. The intervention updated practice to the recommendation of WHO (World Health Organization WHO and the United Nations Children's Fund UNICEF 2020) and the 2023 SSC Research and Practice Guideline (Brimdyr et al. 2023) in which SSC continues uninterrupted for at least 60 min after the birth. This study aims to provide insight into the experience as well as the perceived barriers and solutions faced by health care workers in a regional referral hospital in Uganda as they implement immediate, continuous, uninterrupted skin‐to‐skin contact after birth between mother and baby.

## 2 Methods

### 2.1 Design

A qualitative design was used to evaluate the experience of staff before and after a rapid change in protocol to the implementation of the practice of SSC in the first hour after birth. The full description of the rapid change PRECESS intervention is reported elsewhere (Brimdyr et al. 2024). Semi‐structured key informant interviews were conducted and recorded before and after the change in protocol from usual care (skin‐to‐skin only until the cord was cut) to immediate, continuous uninterrupted skin‐to‐skin practice for all mothers and babies for at least the first hour after birth, except when there was a medical need for separation. The recordings were transcribed and the approach suggested by Vaismoradi et al. was used to code, analyze and develop themes based on the interviews (Vaismoradi et al. 2016).

The current paper describes the analysis of the interviews with hospital staff before and after the intervention, with the aim to understand the staff's experience of the change as well as their thoughts about barriers and solutions to the implementation and continuation of the new protocol, which was not reported elsewhere.

The first group of key informant interviews took place before the intervention, while the staff followed the usual care protocol of SSC only until the cord was cut.

The second set of key informant interviews was conducted 6–7 days after the initiation of the PRECESS intervention aimed at implementing immediate, continuous, uninterrupted skin‐to‐skin contact for the first hour after birth.

### 2.2 Sample and Setting

The study was conducted at a regional training hospital in Uganda, which averages 15–20 births a day. A convenience sample of key‐informant staff members were recruited to be interviewed immediately just before and 6–7 days after the change in care was implemented.

Key‐informants were recruited according to their ability to be absent from their posts. The intention was to include senior leadership, midwives and medical staff, and that was accomplished. To assure anonymity during the analysis, the recordings were labeled according to whether the interview was pre‐ or post‐intervention, the profession of the key‐informant and their number in the interview process. For example, the first midwife to be interviewed pre‐intervention was identified as “Pre‐Intervention Midwife 1.”

Semi‐structured interviews included open‐ended question regarding perceived barriers and solutions to fully implementing SSC at the hospital. They were conducted by two members of the research team, one of whom was Ugandan, in a private room and audio recorded.

### 2.3 Analysis

The key informant audio‐recorded interviews were transcribed. Following the theme construction method described by Vaismoradi et al. transcripts were read by three researchers and meaning units coded. Initial themes were considered, discussed, refined and detailed. Analytical decisions were shared with the full research team who used a consensus process to agree on the final themes as well as the meaning units to be shared in the research report.

### 2.4 Ethical statement

The study was approved by the Makarere School of Health Sciences Research Ethics Committee in Kampala, Uganda (MAKSHSREC‐2023‐558) and the Uganda National Council for Science and Technology (UNCST) registration number HS3183ES. Administrative Permission/Clearance was also provided by Masaka Regional Referral Hospital. All mothers provided informed consent for themselves and their infants to participate in the study.

## 3 Findings

Fifteen semi‐structured in‐depth interviews with key informants were conducted before and after a change in practice to SSC for all mothers and babies in a regional referral hospital in Uganda. The pre‐intervention key informants were interviewed immediately before commencing the intervention and included one medical intern, four midwives and two midwives in senior leadership positions. The post‐intervention interviews had the same format and questions and were conducted 6–7 days later. The eight post‐intervention key informants included two medical interns, three midwives, two obstetricians, and one midwife in a senior leadership position. Only one informant was interviewed in both groups, the lead maternity midwife, who was also a member of senior leadership. The transcribed interviews were analyzed and themes were developed and verified.

Three themes emerged from the analysis of the interviews with leadership and staff:

- Commitment to consistent, evidence‐based patient care within the limits of physical space, supplies and staffing constraints.
- Addressing knowledge and skill regarding the optimal practice of skin‐to‐skin care.
- Willingness to “take up” the change in practice to optimal skin‐to‐skin care for the benefit of mothers and babies.

*Theme #1: Commitment to consistent, evidence‐based patient care within the limits of physical space, supplies and staffing constraints.*

The data presented in this theme indicated that key informants noted limitations of the physical space of the labor and delivery unit, the shortfall of supplies, and the staffing shortages.

The delivery ward has three delivery beds, but only two are considered “preferable” by the staff. The third is higher, and difficult to manage for deliveries. There is also a fourth bed, which is primarily used for triage, but can be used for deliveries if needed. In addition, there are three beds in the “First Stage Room” (for women in labor dilated from 6 to 9 cm), and two beds in admissions.

You see, in the delivery room there, we only have two delivery beds with the other [bed], which is actually not a delivery bed. It is just a bed good for MVA (Manual vacuum Extraction) or just for a repair … perineal tear repair, not for conducting delivery.

(PreIntervention‐Midwife6)

The hospital staff were concerned that keeping a mother for an hour would prevent a bed from being available to the next laboring mother who was ready to give birth.

There are times we get many mothers delivering beyond the beds you have, so if each mother delivers and is given an allowance of one hour, then skin‐to‐skin, you might find some babies, some mothers will deliver on the floor

(PreIntervention‐Midwife2)

The lack of space provides a reason why the staff thought that skin‐to‐skin for the first hour wouldn't be possible. It also posits an insurmountable goal (the need for more beds and more space) that must be achieved before 1 h of SSC would be possible.

The space is too small to accommodate the mothers we have; if we had a bigger space, it (skin‐to‐skin for at least one hour) would be possible.

(PreIntervention‐Midwife5)

Lack of supplies was also perceived as a barrier to implementing skin‐to‐skin in the first hour after birth, especially in the operating theater, where the sterile linens for the operation are the responsibility of the hospital. Before the intervention, the newborn is wrapped on the resuscitation table in the blankets provided by the mother. If they were to remain skin‐to‐skin with the mother on the operating table, additional sterile linens would be required.

In theatre, the linen still is a challenge… Because now there, when they are operating, they have only one sheet to receive the baby, the theater sheet, which they only use because this one is for the baby.

(PreIntervention‐Midwife5)

In addition, the commitment to evidence‐based care raises concerns about staffing. The key informants explained that there are not enough staff already, and that the additional care of providing SSC for an hour with the mother and newborn would push the staff to the limit.

The other challenge is the staff; there are few staff. For example, if interns were not there, the staff is just one; these students are going on a holiday on Friday, and you may not see them. But others might come, but in case they are not around, who will help that mother so that one person has to remove this one and bring it back.

(PreIntervention‐Intern7)

### 3.1 Post‐Intervention

The commitment to evidence‐based care was also evident in the interviews conducted after implementing the change in practice.

After the intervention, space was also described as a limitation. However, now the cited space limitation from before implementation was no longer the beds. The recollection is now that there used to be a lack of space at the resuscitation table, where newborns would be placed during the first hour after birth, and SSC for the first hour had solved this problem.

I think the practice [of skin‐to‐skin contact for the first hour after birth] is going to continue… because we have the limited space, there are times you can find that the resuscitation corner is full, and there is nowhere to put the baby. So, if there is that option of putting the baby immediately skin‐to‐skin for one hour, it will make the immediate skin‐to‐skin work continue.

(PostIntervention‐Intern12)

Supplies were mentioned as a concern during the initial key informant interviews. A sterile cloth for drying and covering the newborn was mentioned as a limitation for implementation of SSC in the operating theater. During PRECESS, which includes reflection and problem solving, it was determined that there are a limited number of sterile cloths that could be used for drying and covering the newborn when skin‐to‐skin. In addition, plans were made to purchase additional cloths specifically for that purpose.

There is no way you can bring a cloth from the community and put it on there… If we have a talk with the theater team, we can have some baby receiving drapes that can be sterilized, and we keep them properly after one hour when properly dressed and washed and sterilized as well I think that would not be a problem.

(PostIntervention‐MD11)

Post intervention, SSC was seen as an advantage to the staffing issue, rather than a reason that it could not be implemented.

Another thing we have seen is that maybe for some time there are no students who act as our assistants sometimes, so if at all you are alone it is easy for you to do skin‐to‐skin and actually you are not overwhelmed by not getting an assistant to take the baby the other side and you can continue working on the mother as the baby is on skin‐to‐skin.

(PostIntervention‐Intern12)

*Theme #2:* Addressing *knowledge and skill regarding the optimal practice of skin‐to‐skin care.*

Interviews with key informants revealed a gap in knowledge for the recommended time of SSC immediately after birth. The amount of recommended time SSC according to the key informants was around 3 min. This coincided with the video recordings of the 92 infants in the pre‐intervention cohort, which revealed an average of two and a half minutes (2:36), and a maximum of 11 min (11:17) (Brimdyr et al. 2024).

I consider skin‐to‐skin contact, then for those 3 minutes… (before the cord is cut).

(PreIntervention‐Midwife3)

When pressed, the key informants mentioned potential time limitations for SSC.

I think the baby shouldn't exceed 30 minutes. On the mother's abdomen, there are hypothermia issues.

(PreIntervention‐Midwife2)

When asked about the possibility of a full hour of SSC pre‐intervention, key informants cited both knowledge and skill gaps.

Maybe some of us are not very aware of the importance of skin‐to‐skin. We need to practice it.

(PreIntervention‐Midwife5)

First‐time mothers presented a specific concern for key informants, since it might be more difficult to breastfeed for the first time laying down, rather than sitting in a chair. This concern highlights a lack of understanding of the newborn's instinctive behaviors while skin‐to‐skin

…for prime gravidas mothers who have delivered for the first time I do not think it would be easy for them … …if the baby is with immediate skin‐to‐skin with the mother for these prima gravidas it will be difficult for them to normally attach the baby onto the breast when she is still lying in that position.

(PreIntervention‐Midwife2)

Skin‐to‐skin contact in the operating theater was also seen as challenge, in this case, due to a skills gap of how it could work.

not in theater because they put that … [drape] that separates the mother's face from what is being done there; even if they put the baby on the tummy, you are under anesthesia at times, you cannot feel the baby properly because of the anesthesia that was given to you…even that cloth that prevents you from seeing what is being done there, how will you help the baby? You will just feel, but you cannot see the baby.

(PreIntervention‐Midwife2)

When babies are placed skin‐to‐skin with their mother during a cesarean surgery, they are traditionally placed above the drape, or above the incision, out of the sterile zone, close to their mother's chest and face. However, the lack of evidence‐based knowledge about the practice lead to a misunderstanding of the practice, and as a result, seeing it as a barrier. There was an understanding that it was possible, but not that it could happen in this hospital.

in developed countries, to say … I have watched some videos where after delivery, the baby is put on the mother's abdomen … skin‐to‐skin … and the baby is left with mother for that one hour, the baby starts breastfeeding from there … even the baby falls asleep with the mother…I think it is possible. It is just because knowledge … we do not have knowledge about it and why we should do for all that long

(PreIntervention‐Midwife2)

### 3.2 Post‐Intervention

After the intervention, the key informants had been practicing the skill of immediate SSC for around an hour immediately after the birth (a mean of 57:52 min). Key informants could explain the skills needed for the procedure, and expressed value in it.

The baby is placed over the chest of the mother and is monitored for the first hour… and this … helps the baby and the mother to unite to be bonded … [it] helps the baby get up well with the temperatures, improving respiration rates and circulation. Yes, it's a very good innovation, and we wish [this] to be done to every child.

(PostIntervention‐Intern8)

It was recognized that what was being taught in school was different from the expected, evidence‐based practice.

All of us in medical school were taught this thing (skin‐to‐skin) … the bonding. When you come to the reality, that's not the way it should be …I cut the cord, I take away the [baby] in theater… this doesn't work at all and also the one hour really hasn't been practiced anywhere.

(PostIntervention‐MD11)

Once immediate, uninterrupted, SSC was implemented, the key informants (and other staff) were able to see the newborn's instinctive behavior. This enhanced knowledge and provided feedback that the skills were being adequately implemented.

my wow moment… the experience are the stages the baby goes through while on the mother's abdomen, yeah, we had not noticed that before because we used to remove babies … we have clamped the cord and cut, we take away the baby. But the time we have left for 1 hour, the stages that the baby goes through is actually something interesting.

(PostIntervention‐Midwife13)

Staff also received feedback about their improved skills by observing changes in the mother's behavior.

There [is] this calmness [the mothers] feel while their babies are with them, they are not worried. Because before, the moment we take the baby, they keep asking us, “Where is my baby … where is my baby?” like their mind is where their baby is. But right now, I think they feel better with their baby and… Even I feel more comfortable when I have the baby and the mother there.

(PostIntervention‐Midwife13)

*Theme #3: Willingness to “take up” the change in practice to optimal skin‐to‐skin care for the benefit of mothers and babies*

One of the necessary elements for the implementation of SSC in the first hour after birth is the willingness of the staff to embrace and move forward with the change in practice. Key informants connected the practice of immediate, continuous, uninterrupted SSC for an hour or more after birth with the benefits for mothers and babies. These benefits included medical rationales, such as mortality:

The team is willing to listen and learn more because we are working towards preserving the lives of the women and our babies.

(PreIntervention‐Leadership1)

as well as psycho‐social rationales, such as bonding.

I do not see the reason why I cannot leave the baby to bond … (or why) I cannot leave the baby together with her mother.

(PreIntervention‐Midwife3)

Key informants could see the connection between skin‐to‐skin contact and patient satisfaction.

I can assure you that when you support them [the mothers], if we support them, they will appreciate and this will happen.

(PreIntervention‐Leadership1)

Even in the operating theater, key informants could see the importance of this new concept of 1 h of skin‐to‐skin contact.

I can say again that yes, even theatre, it will be possible… it is about people understanding why and being willing not to compromise their advantages to the babies and to the mothers.

(PreIntervention‐Leadership1)

Although it could be possible to implement it, the list of challenges felt daunting.

It can be possible … there are issues that you can never stop from happening … especially shortages where you find mothers are many compared to the beds you have, but we can do it…

(PreIntervention‐Midwife3)

### 3.3 Post‐Intervention

After the intervention, key informants shared how they could see the difference in relation to the baby. The strength of the wording—“*we had been depriving the baby of the maternal love”*—emphasizes the strength of the emotion on the part of the key informant.

We have seen that [SSC] is very wonderful, yes, because we had been depriving the baby of the maternal love and bonding and keeping the baby away for some good minutes that we were trying to help the mother in this first hour.

(PostIntervention‐Intern8)

The key informants noticed the difference in the baby's behavior when given more time with the mother.

At first, we were giving this skin‐to‐skin for a few minutes or even seconds, like the maximum would be five. Then we cut the cord and take the baby away. Then, after experiencing this extended period of skin‐to‐skin, I observed that the baby actually becomes more active; it is given room to be active, that is.

(PostIntervention‐Intern9)

And then could connect the behaviors of the baby to the initiation of breastfeeding.

What surprised me was how the baby looked for the breast (all laugh)…wow we have been missing these things, the way it was moving to search… for the breast.

(PostIntervention‐Midwife10)

The key informants also noticed the difference in the mother's behaviors.

It gives a lot of happiness to the mother; the mother feels so good when they are holding their baby on her chest, and it gives them a lot of happiness and joy. They even give you room to do other things; if you are delivering the placenta or closing incisions, they don't mind; they are just looking at their babies. I feel so good when we give extended periods of skin‐to‐skin, and I feel it is beneficial for both the mother and the baby. And I feel we were missing out on those things.

(Post Intern9)

Key informants also connected the changes to their own experience, which helps emphasize the drive to “take up” the procedure.

We have already gotten the skill and we have seen its importance to us, the health workers, and then the mother and the baby because we have been missing it.

(Post Intern8)

Education and the methodology of PRECESS helped health care providers integrate SSC into their work, reinforcing the benefits to the health care provider as well as being best practice.

After the training, people have embraced it. We have liked it personally apart from the few challenges which can be settled.

(PostIntervention‐MD11)

The key informants voice a willingness of the new practice to continue, since there in an understanding of how it meshes with their existing practice, which enhances motivation.

We are going to continue even when you have gone away, you have brought us actually a step to add to what we have been doing.

(PostIntervention‐Midwife14)

The willingness to continue SSC extends beyond the advantages to the mothers and babies, and to the job satisfaction of the health care providers.

Actually, since l started working with [this]. I climb the bed to sleep and sleep deeply because I am satisfied with what l have done. I leave the hospital … excited and satisfied with my job.

(PostIntervention‐Midwife14)

## 4 Discussion

This qualitative study derived three themes from our analysis of 15 interviews of key informant staff members, including midwives, doctors and interns. None of the researchers were hospital employees or worked in the geographic area of Uganda. The interviews were conducted before and after a rapid change intervention (PRECESS) to implement SSC at a regional referral hospital in Masaka, Uganda. We found studies that, like ours, conducted extensive recorded interviews with clinical staff about the barriers to implementing SSC. Koopman et al. report the findings from their interviews of 10 nurses and one medical staff member in the United States (Koopman et al. 2016). Also in the United States, Balatero et al. report on their interviews with 10 nurses who worked in the operating theater (Balatero et al. 2019). Zwedberg et al. report that midwives are “fighting an uphill battle” (p. 215) to expand the implementation of skin‐to‐skin contact according to the eight midwives interviewed in Sweden (Zwedberg et al. 2015). Eighty‐one medical staff and midwives were interviewed or participated in focus groups both before and after a skin‐to‐skin intervention in Gulu, Uganda as reported by Mbalinda et al. (Mbalinda et al. 2018). This study included a SSC intervention as did we, however, they do not report the change in SSC as a result of the intervention. The PRECESS intervention in Masaka Regional Referral hospital resulted in significant improvement of SSC (Brimdyr et al. 2024). Pre‐intervention, the duration of SSC was 2:25 min ± 2:48. Post‐intervention duration of SSC was 57:52 ± 2:43 min (*p* < 0.001), demonstrating effectiveness of the intervention. Six weeks later, the duration of SSC was still high with a mean duration of 58:17 ± 2:02, demonstrating sustainability (Brimdyr et al. 2024).

Our first theme, “Commitment to consistent, evidence‐based patient care within the limits of physical space, supplies and staffing constraints.” found inadequate staffing described as a barrier to implementing SSC before the change in protocol. However, SSC was described a solution to staffing problems after the intervention, since the dyad would remain together. Other constraints that were expressed by staff in our pre‐intervention interviews concerned space and resources. Mbalinda et al. report a concern similar to what we report: “at the current rate of deliveries and current number of delivery beds we have, it is hard unless we have more delivery beds” (Mbalinda et al. 2018) (p. 98). Staff at Masaka Hospital also expressed the concern of insufficient beds and that the 1 h stay for each mother in the birthing bed would cause a back‐up in the busy hospital. This barrier to SSC turned out to be unfounded. Instead, post‐interview, the key informants reported that the mother and newborn staying in the birthing bed was not a staffing or resource issue and, because mothers and newborns were no longer being separated, there was now space on the resuscitation table for medical procedures if needed. Before the intervention babies were separated from their mothers and placed on the table during the third stage of labor, episiotomy repair etc.

Similar to our findings, Koopman et al. and Mbalinda et al. report staffing constraints as a reason that SSC may not or could not be possible (Koopman et al. 2016; Mbalinda et al. 2018). Balatero et al. report that both adequate staffing and workflow in the operating area were a concern “unless we change our whole flow of recovery, it's hard, I think to wait an hour to do things…it just doesn't work in the workflow” (Balatero et al. 2019) (page 140). Koopman et al. expressed a safety issue with implementing SSC. “[what] if the baby is wet and slippery?” (Koopman et al. 2016) (page 1370).

Our key informants expressed the safety concern of maintaining a sterile field when identifying barriers to implementing SSC after Cesarean births. For the operating theater staff members that we interviewed, the concern was very specifically related to material resources: inadequate supplies of sterile cloths to dry and then cover the baby while skin‐to‐skin.

Our second theme is “addressing knowledge and skill regarding the optimal practice of skin‐to‐skin care.” The staff interviewed before the intervention described concerns about new knowledge and skills that would be required to competently practice SSC for at least an hour. Our baseline data indicated that newborns were placed skin‐to‐skin for an under 3 min on average for vaginal births, just until the umbilical cord ceased pulsing and was cut, and not at all for Cesareans (Brimdyr et al. 2024). One staff member in the pre‐intervention group had seen videos of babies in other parts of the world remaining skin‐to‐skin, finding the breast and suckling but admitted that she didn't have the knowledge or skill to attempt prolonged SSC at this hospital. Staff in other studies also expressed concern about needing education before practicing SSC. One participant in the Balatero et al. study combined thoughts about the education needed by their staff with an attitude shift: “it's learning to support skin to skin meaning what's best for mom and baby as opposed to what's best for manpower on the unit…” (Balatero et al. 2019) (p. 140).

Zwedberg et al. report that the midwives they interviewed purported that the reason skin‐to‐skin contact is not prioritized by their co‐workers is that their coworkers were not knowledgeable about the positive effects of the practice (Zwedberg et al. 2015).

Our third theme is the staff's willingness to “take up” the change in practice to optimal skin‐to‐skin care for the benefit of mothers and babies. The driver to sustain the change in practice in our study was expressed as witnessing the positive reactions of the mother and baby and that staff felt happy and satisfied about the change to SSC. One staff member told us that she slept better at night after implementing SSC. Mbalinda et al. also report staff's willingness to continue the new SSC routine. “We kept on doing skin‐to‐skin and we realized it was working out and the mothers were also enjoying it. Because previously, when we separated them, then afterwards you tell her to breastfeed, she says let the baby first be there, I'm tired” (Mbalinda et al. 2018) (p. 99). This quote illustrates the staff's understanding of the connection between SSC and breastfeeding, which was also expressed by the staff in Masaka.

Balatero's interviewees expressed the idea that nurses must continue to advocate for change. One nurse expressed the need for individual advocacy when there isn't system change. “Sometimes people don't pay attention to the latest research supporting SSC, but you just have to. If you feel strong enough, you just have to keep persevering.” (Balatero et al. 2019) (p. 141). The concept of the latest research driving change, rather than the commitment of each individual staff member is reflected by Koopman as well. Koopman and colleague's participants suggested “a clinical algorithm should be established to explicitly define medical conditions of the infant or the mother that contraindicate SSC to exclude any mother–baby dyads at potential risk from early SSC.” (Koopman et al. 2016) (p. 1372) These concepts move the drive for implementation outside of the individual staff members and towards the hospital policies.

Limitations to this study include that the convenience sample included different staff members pre and post intervention. In this way, we sought an impression of the overall unit, rather than changes in a specific individual. This study did not include the impressions of parents before or after the intervention, which could have provided additional insight into the challenges and solutions. Interviews were conducted pre and post intervention, but not at the 6‐week point after the intervention, which could have provided insight into the success of the sustainability of the intervention. Future research is needed to explore the staff's experience of sustaining SSC over time.

## 5 Conclusion

This qualitative study involved semi‐structured interviews with healthcare staff undertaken pre and post‐implementation of a skin‐to‐skin intervention, PRECESS, in a regional referral hospital in Masaka, Uganda. Key informants identified barriers and solutions of staff members as they undertook a change from 3 min of skin‐to‐skin to 1 h of skin‐to‐skin contact after each birth. Analysis of interviews with staff revealed a commitment to consistent, evidence‐based practice in‐line with international guidelines. Key informants addressed the knowledge and skill required to implement the optimal practice of SSC. Despite constraints of the existing environment, staff expressed a willingness to “take‐up” and sustain the practice change for the benefit of mothers and babies. The identified themes provide insight for future implementation of skin‐to‐skin contact.

## Author Contributions

S.N.M., K.B. and K.C. designed the research study. K.B., K.S., M.R., L.R.B., S.N.M., M.K. and A.B. conducted the iterative research and education. S.N.M. and K.B. conducted the interviews. K.C., K.B. and A.B. conducted the thematic analysis and analyzed the data. L.R.B., S.N.M., K.S. and M.K. evaluated the interviews for relationship to the themes and suggested out takes for inclusion. K.C., K.B. and A.B. wrote the first draft of the paper. All authors reviewed, made comments and approved the final draft of the paper.

## Conflicts of Interest

The authors declare no conflicts of interest.

## Data Availability

The data that support the findings of this study are available from the corresponding author upon reasonable request.
